# Bardet-Biedl Syndrome as a Chaperonopathy: Dissecting the Major Role of Chaperonin-Like BBS Proteins (BBS6-BBS10-BBS12)

**DOI:** 10.3389/fmolb.2017.00055

**Published:** 2017-07-31

**Authors:** María Álvarez-Satta, Sheila Castro-Sánchez, Diana Valverde

**Affiliations:** ^1^Grupo de Biomarcadores Moleculares, Departamento de Bioquímica, Genética e Inmunología, Facultad de Biología, Universidad de Vigo Vigo, Spain; ^2^Grupo de Investigación en Enfermedades Raras y Medicina Pediátrica, Instituto de Investigación Sanitaria Galicia Sur (IIS Galicia Sur), SERGAS-UVIGO Vigo, Spain; ^3^Centro de Investigaciones Biomédicas (Centro Singular de Investigación de Galicia 2016–2019), Universidad de Vigo Vigo, Spain

**Keywords:** ciliopathies, chaperonopathies, Bardet-Biedl syndrome, chaperonin-like BBS proteins, MKKS/BBS6, BBS10, BBS12

## Abstract

Bardet-Biedl syndrome (BBS) is a rare genetic disorder that belongs to the group of ciliopathies, defined as diseases caused by defects in cilia structure and/or function. The six diagnostic features considered for this syndrome include retinal dystrophy, obesity, polydactyly, cognitive impairment and renal and urogenital anomalies. Furthermore, three of the 21 genes currently known to be involved in BBS encode chaperonin-like proteins (MKKS/BBS6, BBS10, and BBS12), so BBS can be also considered a member of the growing group of chaperonopathies. Remarkably, up to 50% of clinically-diagnosed BBS families can harbor disease-causing variants in these three genes, which highlights the importance of chaperone defects as pathogenic factors even for genetically heterogeneous syndromes such as BBS. In addition, it is interesting to note that BBS families with deleterious variants in *MKKS/BBS6, BBS10* or *BBS12* genes generally display more severe phenotypes than families with changes in other *BBS* genes. The chaperonin-like BBS proteins have structural homology to the CCT family of group II chaperonins, although they are believed to conserve neither the ATP-dependent folding activity of canonical CCT chaperonins nor the ability to form CCT-like oligomeric complexes. Thus, they play an important role in the initial steps of assembly of the BBSome, which is a multiprotein complex essential for mediating the ciliary trafficking activity. In this review, we present a comprehensive review of those genetic, functional and evolutionary aspects concerning chaperonin-like BBS proteins, trying to provide a new perspective that expands the classical conception of BBS only from a ciliary point of view.

## Bardet-Biedl syndrome in context

The Bardet-Biedl syndrome (BBS; MIM#209900) is a multisystem, rare genetic disorder belonging to the group of ciliopathies, which encompasses several diseases that are caused by defects in cilia structure and/or function, especially affecting the primary cilium (reviewed in Hildebrandt et al., 2011; Mitchison and Valente, 2017). This highly conserved and dynamic organelle is considered the sensorial antennae of the cell and also a central processing unit, since it captures and integrates all the extracellular signals with the cell cycle and metabolism (reviewed in Malicki and Johnson, 2017). Thus, primary cilia play a key role in coordinating the different cellular signaling pathways (reviewed in Cardenas-Rodriguez and Badano, 2009; Christensen et al., 2017), giving rise to biological responses related to the control of cell cycle, development and differentiation processes, migration and polarity, stimuli transduction or proliferation and maintenance of stem cells. Ciliopathies represent an expanding group of human inherited disorders that are valuable models to study several common conditions such as obesity, retinal dystrophy or renal cysts, considering their pleiotropic nature. Remarkably, more than 50 genes have been involved in ciliopathies (Mitchison and Valente, 2017), a number that continues to grow due to the new discoveries on ciliary proteome and ciliogenesis regulation (Mick et al., 2015; Wheway et al., 2015; Boldt et al., 2016), as well as the increasingly implementation of high-throughput sequencing technologies to ciliary disorders. Furthermore, it is important to highlight that ciliopathies are complex clinical entities with extensive genetic heterogeneity and also high phenotypic and genetic overlap among them. This, together with the progressive development of nearly all clinical features related to them, usually makes an early and specific diagnosis very difficult to establish.

BBS is considered a model disease to study the biology of the primary cilium, and is characterized by progressive retinal dystrophy, obesity, postaxial polydactyly, cognitive impairment and renal and urogenital anomalies as primary diagnostic features (reviewed in Forsythe and Beales, 2013). Furthermore, BBS is a genetically heterogeneous disorder with up to 21 genes (commonly known as *BBS* genes) described to date (Bujakowska et al., 2015; Heon et al., 2016; Khan et al., 2016 and references within). Intriguingly, although BBS is primarily inherited as an autosomal recessive disorder, a more complex model of oligogenic inheritance considering modifier *loci* and epistatic effects has been proposed for some families, trying to explain the high clinical variability reported for BBS patients (Katsanis, 2004; Badano et al., 2006). Regarding the functions of BBS proteins (reviewed in Novas et al., 2015), eight of them form a multimeric complex called BBSome, which plays a key role in mediating molecular/vesicular transport in and out of the primary cilium, and also in intraciliary trafficking as part of the intraflagellar transport machinery (Nachury et al., 2007; Loktev et al., 2008; Wei et al., 2012). Moreover, most of the remaining BBS proteins have functions related to BBSome assembly, cilia targeting of BBSome and proper recognition of BBSome cargoes, besides several extra-ciliary roles (Novas et al., 2015).

## Bardet-Biedl syndrome as a chaperonopathy

Three of the main *BBS* genes, *MKKS*/*BBS6* (MIM^*^604896), *BBS10* (MIM^*^610148) and *BBS12* (MIM^*^610683), encode chaperonin-like proteins that localize to centrosomes and ciliary basal bodies (Kim et al., 2005; Marion et al., 2009). This implies that BBS would also be part of the emerging group of diseases called chaperonopathies, which are produced by defects in molecular chaperones or any other protein resembling their structure. In this regard, it is noteworthy that chaperonin-like BBS proteins, as will be explained later, are unlikely to display a folding activity but they have functions specifically related to the assembly of the BBSome.

Chaperonopathies represent an interesting subset of disorders that have so far received little attention, although they can provide useful models to better understand some of the molecular mechanisms necessary to maintain protein homeostasis (extensively reviewed in Macario and Conway de Macario, 2005, 2007a,b; Macario et al., 2005). Chaperonopathies often manifest themselves as complex phenotypes affecting multiple organs, possibly due to the ubiquitous localization of most chaperones, and may be of genetic or acquired origin. In this latter case, defects in chaperone post-translational modifications, distribution or quantity, together with other phenomena such as generation of antichaperone autoantibodies or aggregation of chaperones with deposits of abnormal proteins, all of them usually related to aging, could be the trigger rather than mutational events. Importantly, research on chaperones and their role in disease is opening a new field of therapeutic options (termed “chaperonotherapy”) with interesting applications not only in chaperonopathies, but also in some processes such as cancer whereby chaperones may modulate the immune response against tumors (reviewed in Binder, 2008; Graner et al., 2015).

### Contribution of *MKKS*/*BBS6, BBS10* and *BBS12* genes to Bardet-Biedl syndrome

Among ciliopathies, BBS represents a special case since as far as we know no other ciliopathy except the related McKusick-Kaufman syndrome (MKKS; MIM#236700) is caused by genetic defects in chaperone genes. At this point, it is appropriate to mention that MKKS is a monogenic ciliopathy caused by mutations in the *MKKS* gene leading to postaxial polydactyly, genital malformations (typically hydrometrocolpos in females) and also congenital heart disease (Schaefer et al., 2011). Furthermore, BBS is also a particular member of the chaperonopathies with regard to the very specific functions carried out by chaperonin-like BBS proteins within a ciliary context (explained in detail in the next section). In this sense, BBS constitutes a clear example of the great importance of chaperone defects as determinant pathogenic factors, taking into account that up to 50% of families clinically diagnosed with BBS can harbor pathogenic variants in *MKKS*/*BBS6, BBS10* and *BBS12* genes (Billingsley et al., 2010; Muller et al., 2010; Deveault et al., 2011). This data is even more relevant considering the high genetic heterogeneity of BBS with 21 genes currently identified, a number that is expected to grow as 20–30% of patients suspected to suffer BBS do not yet have molecular confirmation of their clinical diagnosis (Mitchison and Valente, 2017).

**Figure 1:**
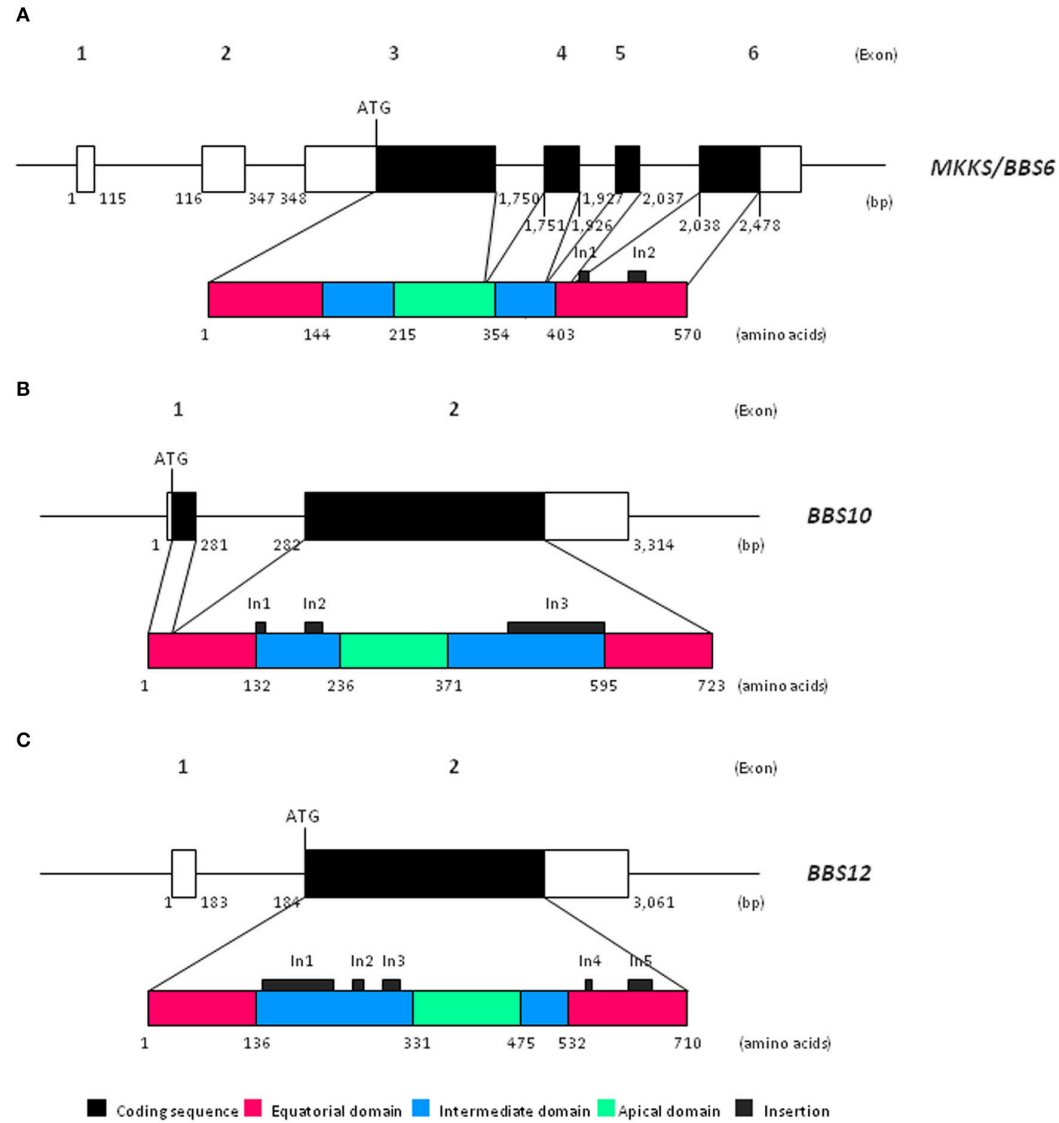
Schematic view of both gene and protein structure of chaperonin-like BBS proteins. **(A)** Representation of *MKKS/BBS6* (reference transcript ENST00000347364.7); **(B)** Representation of *BBS10* (reference transcript ENST00000393262.3); **(C)** Representation of *BBS12* (reference transcript ENST00000314218.7). bp, base pairs.

Chaperonin-like *BBS* genes are characterized by a relatively simple gene structure (Figure 1), with a low number of coding exons (one in *BBS12*, two in the case of *BBS10* and four exons in *MKKS*/*BBS6*), which make them ideal candidates for a mutational screening previously to perform more complex and expensive analyses. Furthermore, a broad distribution of pathogenic variants throughout the coding sequence of chaperonin-like *BBS* genes has been reported. A brief summary of the most relevant genetic findings concerning each gene is presented below (see also Table 1).

**Table 1:** Summary of the main features related to chaperonin-like *BBS* genes.

**Gene**	**Gene MIM number**	**Chromosome**	**Exons (Coding)**	**Pathogenic variants^†^**	**Mean contribution^∧^(%)**	**Protein (aa)**	**Phenotype MIM number**	**References**
*MKKS/BBS6*	^*^604896	20p12.2	6 (4)	57	3–5	570	#605231 (BBS) #236700 (MKKS)	Katsanis et al., 2000 Slavotinek et al., 2000; Stone et al., 2000
*BBS10*	^*^610148	12q21.2	2 (2)	99	20	723	#615987 (BBS)	Stoetzel et al., 2006
*BBS12*	^*^610683	4q27	2 (1)	59	8–11	710	#615989 (BBS)	Stoetzel et al., 2007

*MIM, Mendelian Inheritance in Man® (online database of human genes and genetic diseases; https://www.omim.org/); aa, amino acids; BBS, Bardet-Biedl syndrome; MKKS, McKusick-Kaufman syndrome*.

† *The number of pathogenic variants corresponds to the data obtained from the last version of the Human Gene Mutation Database (HGMD professional 2017.1; released on March 2017)*.

∧ *The mean contribution for each chaperonin-like BBS gene was established from the values reported elsewhere (Beales et al., 2001; Stoetzel et al., 2006; Billingsley et al., 2010; Muller et al., 2010; Deveault et al., 2011; Forsythe and Beales, 2013; Álvarez-Satta et al., 2014)*.

*MKKS*/*BBS6* (chromosome 20p12.2) was the first gene coding a putative chaperonin to be associated with a human inherited disorder, the MKKS (Stone et al., 2000), being also involved in BBS shortly after (Katsanis et al., 2000; Slavotinek et al., 2000). To date, more than 50 deleterious variants have been described, predominantly missense and nonsense changes (Human Gene Mutation Database; Stenson et al., 2017). Regarding its contribution to the total load of BBS, *MKKS*/*BBS6* is a minor contributor with 3–5% of families harboring two disease-causing variants in the multiethnic cohorts reported worldwide (Beales et al., 2001; Muller et al., 2010; Deveault et al., 2011). Interestingly, the vast majority of causal variants described in this gene have been identified in BBS patients, so it has been proposed that both syndromes MKKS and BBS are different allelic forms of the same clinical entity (Katsanis et al., 2000; Schaefer et al., 2011). Thus, MKKS phenotypes would be linked to very rare, possibly hypomorphic alleles found in *MKKS*/*BBS6* gene.

The *BBS10* gene (chromosome 12q21.2) was first identified by Stoetzel et al. (2006) in a consanguineous pedigree of Lebanese origin. It is, together with *BBS1*, the major contributor to BBS accounting for 20% of all cases (Stoetzel et al., 2006; Forsythe and Beales, 2013), with remarkable exceptions in ethnically homogeneous groups such as Danish (43%; Hjortshøj et al., 2010) or Spanish BBS cohorts (8.3%; Álvarez-Satta et al., 2014). About 100 different disease-causing changes have been reported elsewhere (Human Gene Mutation Database; Stenson et al., 2017), of which the p.Cys91Leufs^*^5 allele represents a recurrent deleterious variant in BBS cohorts of European descent, reaching 26–48% of *BBS10* mutational load (Stoetzel et al., 2006; Billingsley et al., 2010; Muller et al., 2010).

Moreover, the *BBS12* gene (chromosome 4q27) was linked to BBS phenotypes a decade ago (Stoetzel et al., 2007). Its contribution to BBS has grown in importance over recent years, accounting for 8–11% of the total cases in most of the cohorts reported (Billingsley et al., 2010; Muller et al., 2010; Deveault et al., 2011; Álvarez-Satta et al., 2014). About 60 pathogenic variants have been currently identified in *BBS12* patients (Human Gene Mutation Database; Stenson et al., 2017), among which the nonsense change p.(Phe372^*^) could represent up to 20% of the mutated alleles found in this gene (Stoetzel et al., 2007).

Finally, it is also important to highlight some trends regarding the BBS phenotypes linked to changes in chaperonin-like *BBS* genes. Thus, there is a general consensus that BBS patients with pathogenic variants in *MKKS/BBS6, BBS10* and *BBS12* genes develop a more severe phenotype than those with changes affecting BBSome components such as BBS1 (Billingsley et al., 2010; Imhoff et al., 2011; Castro-Sánchez et al., 2015). In detail, they show an earlier disease onset (especially noted in *BBS10* patients), greater prevalence of all BBS primary diagnostic features and also a higher frequency of overlapping features with other ciliopathies, mainly MKKS and also Alström syndrome (MIM#203800), which is a closely related ciliopathy produced by mutations in the *ALMS1* gene and characterized by retinal dystrophy, sensorineural hearing loss, early-onset obesity with severe type 2 diabetes mellitus and metabolic syndrome, dilated cardiomyopathy and renal, hepatic and pulmonary injury with widespread fibrosis (reviewed in Marshall et al., 2011). One could hypothesize that differences in the severity of clinical presentation could be due to the distinct functional roles of chaperonin-like *BBS* genes when compared with the BBS proteins taking part of the BBSome. Thus, deleterious variants in some components of the BBSome might lead to the accumulation of intermediate complexes that maintain a residual or gain-of-function activity as compensating mechanism (Zhang et al., 2012), whereas the chaperonin-like BBS proteins are essential for the initial step of BBSome assembly (see below) so no functional complexes are formed if this subset of proteins is affected (Seo et al., 2010).

### Structure and function of chaperonin-like BBS proteins: comparison with canonical CCT chaperonins

The three chaperonin-like BBS proteins define a particular branch of proteins that have sequence homology to the chaperonin containing t-complex protein 1, CCT (also known as TRiC) family of group II chaperonins (Kim et al., 2005; Stoetzel et al., 2006, 2007). CCT proteins are the eukaryotic cytosolic chaperonins of type II and play key roles in the folding of a wide range of newly translated proteins in an ATP-dependent manner, mainly soluble proteins related to cytoskeleton (actin and tubulin are the quantitative major substrates) (reviewed in Dunn et al., 2001; Spiess et al., 2004). Typically, they form a functional hetero-oligomeric complex of 16 subunits that consists of two stacked rings, each composed of eight CCT monomers radially arranged (CCT1-8). With regard to their specific roles in cilia, CCT subunits are required for ciliary assembly and maintenance of cilia tip integrity, as well as cytoskeleton structure, in the ciliate *Tetrahymena* (Seixas et al., 2010). In addition, it has been recently reported that CCT chaperonins are essential for the biogenesis of vertebrate photoreceptors' outer segment by mediating the BBSome assembly (Sinha et al., 2014).

Recent phylogenetic analyses have revealed that chaperonin-like BBS proteins represent a highly diverged, monophyletic group derived from a duplication event in the *CCT8* gene (Mukherjee et al., 2010). Remarkably, although *MKKS/BBS6, BBS10*, and *BBS12* genes were originally considered as vertebrate-specific, the finding of several orthologs in ancient eukaryotes clearly points to an earlier evolution (Mukherjee and Brocchieri, 2013). The high rate of divergence observed for chaperonin-like BBS proteins compared with those canonical CCT chaperonins is not reflected by their primary structure, which is mostly conserved. Thus, the typical chaperonin domain architecture consisting of apical, intermediate and equatorial domains is conserved in chaperonin-like BBS proteins (Figure 1); however, they have additional specific insertions (two in MKKS/BBS6, three for BBS10 and up to five in the BBS12 sequence) that are restricted to intermediate and equatorial domains (Kim et al., 2005; Stoetzel et al., 2006, 2007). Interestingly, the three insertions located in BBS10, as well as the insertions 1 and 3 of BBS12, protrude from the same face of the intermediate domain, which suggests they constitute an additional domain maybe with specific roles (Stoetzel et al., 2006, 2007). Furthermore, BBS12 seems to be the most divergent member since more differences in several secondary-structure motifs and also in the ATP-hydrolysis motif have been identified (Stoetzel et al., 2007; Mukherjee et al., 2010).

Despite structural similarities, solid evidences point out that chaperonin-like BBS proteins neither perform folding activity nor are able to form chaperonin oligomeric complexes like canonical CCT proteins do. Thus, the ATP hydrolysis motif in the equatorial domain (highly conserved in Group I and II chaperonins) is significantly different in MKKS/BBS6 and, above all, in BBS12 protein (Kim et al., 2005; Stoetzel et al., 2007), which suggests that the catalytic activity required for protein folding is missing; conversely, it would be conserved in BBS10 (Stoetzel et al., 2006). In addition, the existence of specific insertions in chaperonin-like BBS proteins covering potential monomer-monomer contact regions makes it unlikely that they can assemble in a functional CCT-like complex (Kim et al., 2005; Stoetzel et al., 2006, 2007; Mukherjee et al., 2010).

All these data suggest that the roles of chaperonin-like BBS proteins may differ from direct protein folding. Thus, recent work has demonstrated that MKKS/BBS6, BBS10 and BBS12 play a key role in the initial steps of BBSome assembly by stabilizing BBS7 (the first component to be incorporated) and mediating its interaction with six canonical CCT chaperonins (CCT1-5 and CCT8), which would actually accomplish the folding activity (Seo et al., 2010). This means that chaperonin-like BBS proteins act as an intermediate for the binding of CCT complex to its substrates, as part of the transient BBS/CCT/TRiC-chaperonin complex. Remarkably, BBS10 is not a structural member of this complex, but it regulates the interaction of the BBS6-BBS12-BBS7 intermediate with CCT proteins to form the BBS/CCT/TRiC-chaperonin complex (Zhang et al., 2012). It is also important to note that the second step in BBSome assembly, that is, the interaction between BBS2 and the stabilized BBS7 protein, is coupled with the release of BBS6-BBS12 from the complex, and that CCT/TRiC proteins are also released after the BBSome core complex (BBS2-BBS7-BBS9) is formed (Zhang et al., 2012). Accordingly, the BBS/CCT/TRiC-chaperonin complex would assist BBSome assembly only in the first steps, so the formation of mature BBSome complexes is finally completed by intrinsic protein-protein interactions among the BBSome components, which are known to contain β-propeller, tetratricopeptide repeats and pleckstrin homology domains that typically mediate these interactions (Zhang et al., 2012). Despite the significant progress made in deciphering the specific roles of chaperonin-like BBS proteins, details on how the BBS/CCT/TRiC-chaperonin complex is formed and completes the transition of BBS7 to BBSome remain to be elucidated.

### Role of other BBS proteins in protein homeostasis

The cellular network for protein-quality control necessary to maintain protein homeostasis includes besides the chaperone machinery, which ensures proper protein folding and recognition of misfolded proteins (reviewed in Hartl et al., 2011), also two proteolytic machineries, the ubiquitin-proteasome system and the autophagy pathway, which play essential roles in removing irreversibly misfolded proteins (reviewed in Chen et al., 2011). In this regard, it is appropriate to remark on some findings involving several BBS proteins and their possible role in this field.

Several BBSome components such as BBS1-2, BBS4, and BBS7, as well as the BBS6 chaperonin-like protein, interact with proteasomal subunits and could be involved in the regulation of signaling pathways coordinated by the primary cilium (reviewed in Novas et al., 2015). It has been also speculated that TRIM32/BBS11 (MIM^*^602290) would be the putative E3-ubiquitin ligase that targets free BBS2 to be degraded by the ubiquitin-proteasome pathway (Zhang et al., 2012). Finally, there is also evidence that the *unfolded protein response* (UPR) of the endoplasmic reticulum can be a pathogenic mechanism related to BBS, as the UPR is triggered by protein accumulation in the photoreceptors of *Bbs12*-deficient models leading to apoptosis and subsequent retinal degeneration (Mockel et al., 2012). Interestingly, the light detection ability was restored by pharmacological modulation of the UPR, which highlights both the importance of identifying disease mechanisms that involve proteostasis network components and their potential to develop new therapeutic strategies.

## Perspectives

BBS is considered a model ciliopathy to study molecular mechanisms potentially involved in common disorders such as obesity, and also represents a singular component of the group of chaperonopathies. Unlike many of these clinical entities, the molecular basis underlying BBS is fairly well-known, just like the particular role of most BBS proteins in the primary cilium and also the cellular basis of several BBS phenotypes (reviewed in Novas et al., 2015). However, many mechanistic aspects remain to be uncovered, especially those concerning the particular molecular processes involved in initialization of BBsome assembly and also the role of protein degradation systems in BBS proteins turnover. In this sense, the use of prokaryotic chaperonins as models to investigate the impact of deleterious variants in chaperonin structure and function, as well as potential therapeutic strategies (Conway de Macario et al., 2017), could represent a promising tool not explored until now to further characterize chaperonin-like BBS proteins.

Moreover, a deeper understanding of the molecular mechanisms involving chaperonin-like BBS proteins could provide more opportunities to explore new therapies for BBS patients, currently unavailable. Thus, some BBSome components are found in monomeric form or aggregated with unidentified proteins in *Bbs6* null mice (Seo et al., 2010), which might suggest potential therapeutic targets related to the modulation of chaperone activity. In addition, identifying the specific chaperones and partners involved in the folding of BBSome components, not yet defined, could have a great impact on the development of new strategies in this field.

## Author contributions

MA conceptualized the study, and drafted, reviewed and edited the manuscript. SC drafted and reviewed the manuscript. DV conceived of the study, critically revised the manuscript, acquired funds and supervised the work. All authors read and approved the final manuscript.

### Conflict of interest statement

The authors declare that the research was conducted in the absence of any commercial or financial relationships that could be construed as a potential conflict of interest.
